# Age-Dependent Sex Bias in Clinical Malarial Disease in Hypoendemic Regions

**DOI:** 10.1371/journal.pone.0035592

**Published:** 2012-04-25

**Authors:** Sulabha Pathak, Mayuri Rege, Nithya J. Gogtay, Umesh Aigal, Surya Kant Sharma, Neena Valecha, Gyan Bhanot, Nilima A. Kshirsagar, Shobhona Sharma

**Affiliations:** 1 Department of Biological Sciences, Tata Institute of Fundamental Research, Mumbai, India; 2 Department of Clinical Pharmacology, Seth GS Medical College, KEM Hospital, Mumbai, India; 3 Kasturba Hospital for Infectious Diseases, Mumbai, India; 4 National Institute of Malaria Research, Field station, Odisha, India; 5 National Institute of Malaria Research (ICMR), New Delhi, India; 6 Department of Molecular Biology and Biochemistry, Department of Physics, Rutgers University, Piscataway, New Jersey, United States of America; Université Pierre et Marie Curie, France

## Abstract

**Background and Objectives:**

Experimental models show a male bias in murine malaria; however, extant literature on biases in human clinical malaria is inconclusive. Studies in hyperendemic areas document an absence of sexual dimorphism in clinical malaria. Data on sex bias in clinical malaria in hypoendemic areas is ambiguous—some reports note a male bias but do not investigate the role of differential mosquito exposure in that bias. Moreover, these studies do not examine whether the bias is age related. This study investigates whether clinical malaria in hypoendemic regions exhibits a sex bias and whether this bias is age-dependent. We also consider the role of vector exposure in this bias.

**Methods:**

Retrospective passive clinical malaria datasets (2002–2007) and active surveillance datasets (2000–2009) were captured for the hypoendemic Mumbai region in Western India. To validate findings, passive retrospective data was captured from a primary malaria clinic (2006–2007) in hypoendemic Rourkela (Eastern India). Data was normalized by determining percent slide-positivity rates (SPRs) for males and females, and parasite-positivity distributions were established across age groups. The Mann–Whitney test, Wilcoxon Signed Rank test, and Chi-square analysis were used to determine statistical significances.

**Results:**

In both the Mumbai and Rourkela regions, clinical malaria exhibited an adult male bias (p<0.01). A sex bias was not observed in children aged ≤10. Post-puberty, male SPRs were significantly greater than females SPRs (p<0.01). This adult male bias was observed for both vivax and falciparum clinical disease. Analysis of active surveillance data did not reveal an age or sex bias in the frequency of parasite positivity.

**Conclusion:**

This study demonstrates an age-dependent sex bias in clinical malaria in hypoendemic regions and enhanced incidence of clinical malaria in males following puberty. Possible roles of sex hormones, vector exposure, co-infections, and other factors in this enhanced susceptibility are discussed.

## Introduction

Malaria accounts for a major portion of the global disease burden [1]. A male bias in malarial infections has been noted in host species as diverse as lizards and great tits [2], [3]. In the absence of vector-exposure data, it is unclear if the observed male bias is a consequence of differential mosquito exposure. However, experimental models, which allow for controlled exposure, are useful in bias-determining studies. Studies in the murine model clearly establish a male bias in malaria and show that testosterone increases susceptibility to the disease [4], [5]. Information regarding a sex bias in human malarial susceptibility is inconclusive. The pattern of age-specific malaria morbidity is well established in hyperendemic areas, where the incidence of clinical malaria peaks in childhood [6]–[9]. Children between the ages of 5 and 10 develop immunity to severe disease while continuing to suffer from mild disease. Even in adulthood, sterile immunity to infection is never acquired, and adults living in hyperendemic areas rarely suffer from clinical disease even though they often carry the parasite [10], [11]. Sexual dimorphism does not exist in hyperendemic regions for both *Plasmodium falciparum* and *P. vivax* infections, although some reports note an increased parasite density in pubertal and post-pubertal males [7], [12]. The only occurrence of a sex bias in hyperendemic areas is observed in women in their first pregnancy, who have an increased risk of falciparum malaria; this is attributed to the ability of the parasite to sequester in the placenta [13], [14].

The data on sex bias in hypoendemic areas is equivocal. A few studies observed a skewed male∶female ratio in clinical malaria but did not investigate the phenomenon [15]–[18]. Instead, the bias was attributed to differential mosquito exposure—the outdoor patterns of activity in these regions were thought to expose more males than females to infected mosquito bites [16]–[18]. This study was undertaken to determine whether clinical malaria exhibits a sex bias in hypoendemic regions and if such bias is age-dependent. Active surveillance data which might act as an indicator for vector-exposure, was also analyzed.

The study was conducted primarily in the hypoendemic urban area of Mumbai, situated in Western India. In this region, the principal malarial vector is the indoors- and outdoors-biting *Anopheles stephensi* [19]; the predominant malarial parasite is *Plasmodium vivax* [20]. Retrospective active surveillance records were used as an indicator of vector exposure. Age and sex distributions of clinical malaria were assessed from passive data obtained from both high- and low-cost medical centers. Analysis of data from Mumbai suggested that clinical malaria exhibited an adult male bias; sex bias was not observed in children under ten years of age. We substantiated our conclusion by analyzing data from Rourkela, another urban hypoendemic region in Odisha, on the Eastern side of the country. The predominant parasite in Rourkela is *P. falciparum*, which is spread by *An. culicifacies* [21], [22]. This study presents data indicating an age-dependent sex bias in human clinical malaria in hypoendemic regions that is not reflected in the active surveillance records and hence is likely not attributable to differential mosquito exposure.

## Methods

### Ethics statement

The retrospective data was collected and analyzed anonymously, free of any personally identifiable information. Therefore, informed consent was not necessary. Data was collected after obtaining appropriate institutional clearances as required by the Ethics Committee for Research on Human Subjects of Seth GS Medical College and KEM Hospital, Mumbai, and National Institute for Malaria Research.

### Study area

**Figure 1:**
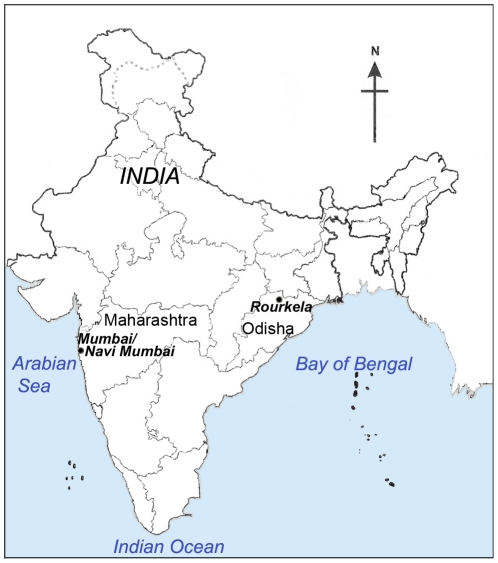
Geographical location of data collection sites in India.

This retrospective study was conducted in the urban Mumbai region of Western India, consisting of Mumbai and Navi Mumbai (Figure 1). The two interconnected, conurbated cities are separate entities for administrative rather than geographical reasons. To validate findings, data was also collected from the city of Rourkela, Odisha, another hypoendemic region situated in Eastern India (Figure 1). The malaria endemicity of the town of Rourkela is similar to that of the deforested plain areas in Sundargarh district [22]. Thus, by to the WHO-accepted Metselaar & Van Thiel classification, both these regions are malaria hypoendemic, as <10% of children aged 2–9 years show presence of the parasite in their peripheral blood [20]–[23].

### Malarial diagnosis

Diagnosis of clinical malaria was based on presenting symptoms and parasite detection. Parasite identification was done by microscopic examination of Giemsa-stained thick and thin blood smears. Blood was obtained by venous puncture or finger-prick. The data was normalized by determining percent slide-positivity rates (SPRs) for both *P. vivax*, and *P. falciparum*.
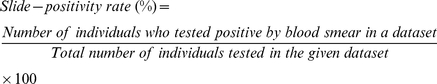



### Passive clinical malaria data

Records from public and private medical centres for the years 2002–2007 provided the clinical malaria dataset for the Mumbai region. It consisted of 31,616 patients who presented with malaria-like symptoms (fever, chills, headache, etc.) at the King Edward Memorial (KEM) Hospital and Kasturba Hospital for Infectious Diseases in Mumbai, and private medical centres in Navi Mumbai. Relapsed/recrudescent patients, repeat blood samples, and pregnant women were excluded from analysis. Most malarial patients (>80%) were treated in the ‘Out Patients Department’ and did not require hospitalization. Both KEM and Kasturba hospitals are government funded and provide access to low-cost medical services. To test for biases caused by under-reporting due to economic considerations, data was also collected from private medical centres in Navi Mumbai that offer higher-cost medical services. Additionally, data was also collected from a government-run free primary malaria referral clinic in Rourkela (2006 and 2007). Majority of the patients reporting to this referral clinic belong to the town and peripheral areas of Rourkela city. Parasite-positive patients from this passive dataset constituted clinically diseased individuals seeking medical intervention for their malarial infection.

### Active Surveillance Data

The malaria surveillance and control program is a house-to-house survey designed to detect suspected cases of malaria that are subsequently confirmed by blood-smear analysis; as per government policy, all positive cases are immediately treated with anti-malarial drugs. The main purpose of this surveillance is to detect changes in trends or distribution in malaria in order to initiate investigative or control measures. Under the malaria surveillance and control program, municipal health workers routinely visit residential areas, slums, construction sites, and municipal schools every fortnight. The incubation interval for *P. vivax* is ∼22 days whereas for *P. falciparum* it is 35 days. A surveillance cycle of 15 days (less than one incubation interval) is designed to catch most of the secondary cases before the commencement of next cycle [24]. The health workers collect blood smears from individuals who report fevers, headaches, or other potentially malaria-associated symptoms during the visit or the preceding fortnight. Smears are also requested of family members and immediate neighbors. This allows the workers to find the source of infection, identify all cases and susceptible contacts as well as others at risk, and treat the parasite-positive individuals with appropriate anti-malarial drugs. Detailed active surveillance records yielding age and sex distributions of the monitored population could not be obtained from the municipal governance body for Mumbai city, but the municipal governance body of Navi Mumbai provided such comprehensive active surveillance records. Parasite-prevalence in the general populace was ascertained from the registers of three such malaria surveillance centers—Urban Health Projects 13, 15, 16—for the years 2000–2009. The records of 21,924 individuals thus obtained formed the active surveillance dataset. Parasite-positive persons from this dataset represent individuals who were asymptomatic or whose symptoms did not necessitate hospital-based medical intervention. Active surveillance has been discontinued in Rourkela from June 2007. The combined number of individuals surveyed in 2006 and 2007 were 3998, and therefore, too small for detailed analysis. The active and passive datasets were non-overlapping. The collated clinical malaria and active surveillance data used in this study is publicly available online at http://www.tifr.res.in/~dbs/faculty/sslab/index_files/epidem_data.htm.

### Statistical analysis

Statistical analysis was carried out using GraphPad Instat [25]. In keeping with groupings from previous literature, active and passive datasets were analyzed in 15-year age bins. To assess the influence of season, the active and passive datasets were segregated into two groups for analysis—the wet Monsoon (June to September) and dry Non-monsoon (October to May) seasons. To enable statistical analysis, the two-year Rourkela data was divided into four six-month units (January–June and July–December). The test statistic used for analysis was SPR. Because the SPR is a ratio, it does not suffer from ascertainment bias, i.e., the fraction of males or females that have malaria remains unchanged (except for statistical fluctuations), even if there should be a bias towards testing more individuals of a given sex.

The mean age of menarche for Indian girls has been variously reported to be from age 12.4 to 13.1 years [26], [27], and the age at which Indian women enter menopause is estimated to range from 35–51 years [28], [29]. Age of puberty of Indian boys is considered to be >11 years [30]. Hence, to test the relationship, if any, between puberty, reproductive years, and clinical malaria, parasite-positivity distribution patterns were assessed in ≤10, 20–40, and >55 age groups. SPR data was also transformed to determine the fraction of positives contributed by males and females in a particular class. Transformed and untransformed datasets were analyzed using the non-parametric Mann–Whitney test and the Wilcoxon Signed Rank test. Active surveillance data for *P. falciparum* had too few points for the Wilcoxon Signed Rank test, and as a result, this test could not be applied to that dataset. Passive datasets across years for Mumbai as well as for Rourkela were combined and the two combined datasets analyzed by Chi-square/Fisher's exact test. Mixed infections were rare in both datasets (<0.5% in Mumbai, <1.5% in Rourkela), and these were therefore included in analysis. Statistical significances remained unchanged when the mixed infection data was excluded from analysis.

## Results

### Pattern of parasite species distributions

Table 1 shows retrospective data of patients in the Mumbai passive clinical malaria dataset. Of the 31,616 patients who approached medical centers for their malaria-like symptoms, 2,948 (9.3%) tested positive for the parasite. In concordance with previous reports, *P. vivax* was found to be the predominant parasite, accounting for ∼76% (range 69.5–88.7%) of clinical malaria patients [15]. *P. falciparum* accounted for the remaining ∼24% (range 11.3–30.5%). Clinical disease caused by mixed infections was rare; the dataset includes only 18 such instances.

**Table 1:** Passive clinical malaria datasets.

Source	Total checked				*P. vivax* positive (SPR)				*P. falciparum* positive (SPR)			
	Males		Females		Males		Females		Males		Females	
	≤15	>15	≤15	>15	≤15	>15	≤15	>15	≤15	>15	≤15	>15
**Mumbai region**												
KEM Hospital 2002	392	3130	259	1386	11 (2.8)	335 (10.7)	8 (3.1)	74 (5.3)	3 (0·8)	105 (3.4)	4 (1.5)	16 (1.2)
KEM Hospital 2003	376	3142	193	1380	27 (7.2)	324 (10.3)	12 (6.2)	60 (4.3)	15 (4.0)	148 (4.7)	3 (1.6)	20 (1.4)
KEM Hospital 2004	469	3040	282	1156	16 (3.4)	338 (11.1)	15 (5.3)	52 (4.5)	10 (2.1)	62 (2.0)	3 (1.1)	6 (0.5)
KEM Hospital 2005	654	3851	360	1654	9 (1.4)	179 (4.6)	6 (1.7)	32 (1.9)	3 (0.5)	26 (0.7)	2 (0.6)	4 (0.2)
Kasturba Hospital	1445	5567	746	1268	65 (4.5)	443 (8.0)	18 (2.4)	64 (5.0)	18 (1.2)	196 (3.5)	4 (0.5)	26 (2.1)
Private medical centers	46	528	25	267	2 (4.3)	134 (25.4)	0 (0.0)	29 (10.9)	1 (2.2)	19 (3.6)	0 (0.0)	1 (0.4)
Total	3382	19258	1865	7111	130 (3.8)	1753 (9.1)	59 (3.2)	311 (4.4)	50 (1.5)	556 (2.9)	16 (0.9)	73 (1.0)
**Rourkela**												
2006	3181	6112	2105	2734	34 (1.1)	151 (2.5)	21 (1.0)	31 (1.1)	60 (1.9)	366 (6.0)	55 (2.6)	135 (4.9)
2007	2832	6008	1989	2819	46 (1.6)	170 (2.8)	30 (1.5)	49 (1.7)	82 (2.9)	333 (5.5)	76 (3.8)	112 (4.0)
Total	6013	12120	4094	5553	80 (1.3)	321 (2.6)	51 (1.2)	80 (1.4)	142 (2.4)	699 (5.8)	131 (3.2)	247 (4.4)

In the active surveillance dataset from Mumbai, the parasite was detected in 257 individuals out of 21,924 persons tested (Table 2). This parasite detection rate of 1.17% (range 0.07–4.6%) was found to be in agreement with previous reports for the region [15], [20]. Only one parasite positive individual harbored both parasites. The annual detection rates of *P. vivax* and *P. falciparum* ascertained from the active surveillance records were between 0.07–2.5% and 0–2.14% respectively. The age and sex distributions of individuals in the passive and active datasets as well as of those who tested positive for *P. vivax* and/or *P. falciparum* are shown in Table 1 and 2 respectively.

**Table 2:** Active surveillance datasets (Mumbai region).

	Total checked				*P. vivax* positive (SPR)				*P. falciparum* positive (SPR)			
	Males		Females		Males		Females		Males		Females	
Year	≤15	>15	≤15	>15	≤15	>15	≤15	>15	≤15	>15	≤15	>15
2000	339	771	322	534	2 (0.6)	10 (1.3)	0 (0.0)	3 (0.6)	0 (0.0)	7 (0.9)	2 (0.6)	0 (0.0)
2001	226	599	208	366	4 (1.8)	14 (2.3)	5 (2.4)	12 (3.3)	5 (2.2)	15 (2.5)	3 (1.4)	7 (1.9)
2003	509	860	457	882	10 (2.0)	6 (0.7)	6 (1.3)	7 (0.8)	2 (0.4)	8 (0.9)	1 (0.2)	4 (0.5)
2004	635	1129	559	1025	5 (0.8)	15 (1.3)	7 (1.3)	4 (0.4)	2 (0.3)	1 (0.1)	0 (0.0)	2 (0.2)
2005	910	2018	772	1466	3 (0.3)	25 (1.2)	4 (0.5)	11 (0.8)	2 (0.2)	10 (0.5)	2 (0.3)	1 (0.1)
2006	80	594	62	371	0 (0.0)	9 (1.5)	1 (1.6)	0 (0.0)	0 (0.0)	1 (0.2)	0 (0.0)	0 (0.0)
2007	196	1341	145	682	0 (0.0)	9 (0.7)	2 (1.4)	2 (0.3)	0 (0.0)	0 (0.0)	0 (0.0)	0 (0.0)
2008	133	626	107	285	1 (0.8)	2 (0.3)	0 (0.0)	1 (0.4)	0 (0.0)	0 (0.0)	0 (0.0)	0 (0.0)
2009	231	1505	193	786	1 (0.4)	1 (0.1)	0 (0.0)	0 (0.0)	0 (0.0)	0 (0.0)	0 (0.0)	0 (0.0)
Total	3259	9443	2825	6397	26 (0.8)	91 (1.0)	25 (0.9)	40 (0.6)	11 (0.3)	42 (0.4)	8 (0.3)	14 (0.2)

The Rourkela passive data was collected to test our hypothesis regarding an adult male bias in clinical malaria in hypoendemic regions. A total of 27,780 individuals approached the primary malaria clinic in Rourkela for their malaria-like symptoms during 2006 and 2007. Of these, 1,751 (6.30%) tested positive for the parasite (Table 1). As reported earlier [22], *P. falciparum* was the predominant parasite, accounting for 69.6% of the infections whereas 30.4% tested positive for *P. vivax*. There were 17 instances of mixed infections. Active surveillance has been discontinued in Rourkela from July 2007. The SPR based on the data available was ∼2.18% (data not shown).

### Age and sex distributions of parasite-positivity in passive and active datasets

**Figure 2:**
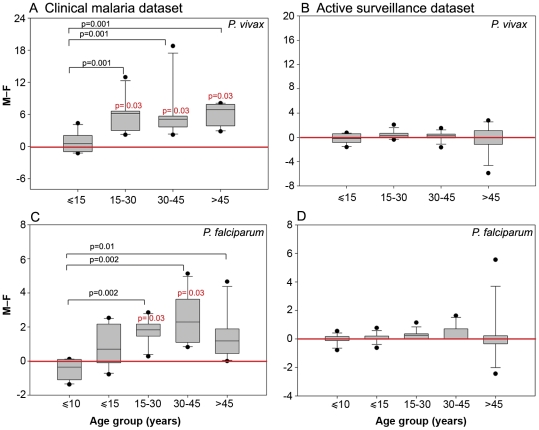
Age distributions of differences between male and female SPRs in the clinical malaria and active surveillance datasets in the Mumbai region. A,C, Box plot showing the 25th and 75th percentiles, together with the median, with whiskers showing the minimum and maximum difference in the percent slide-positivity rates between males and females across age groups in the clinical malaria dataset for vivax (A) and falciparum (C) malaria. B,D, Box plots as in A showing the difference in the percent slide-positivity rates between males and females across age groups who tested positive for *P. vivax* (B) and *P. falciparum* (D) in the active surveillance programme. Data were compared with the difference of male/female SPRs expected under the hypothesis of neutrality (0, red line) and were analyzed with the Mann–Whitney test. Statistically significant values are shown in black. Numbers in red indicate statistically significant p values obtained by Wilcoxon Signed Rank test under the hypothesis that the median of the group did not differ significantly from zero. The test could not be applied to the falciparum data in the active surveillance dataset.

To assess the existence of a sex bias, we determined the difference between male and female SPRs. SPR is a ratio that represents the percent of malaria positive individuals in a given category and does not suffer from ascertainment bias even if greater number of individuals of a particular sex are included in a given group. Under the hypothesis of neutrality, the difference between male and female SPRs should be distributed around zero. In the Mumbai region, a sex bias was found in the clinical malaria dataset but not in the active surveillance dataset (Figure 2). This was true for both species of the parasite.

We found that clinical malaria was primarily an adult male disease. Although greater number of males than females reported to medical facilities for their malaria-like symptoms (69% males compared to 31% females, Table 1), the use of SPRs as the test statistic and the different types of analyses performed ensured that this preponderance of males did not affect our conclusions. Sex bias was absent in children under age 15 in the case of vivax malaria (Figure 2A; Mann–Whitney and Wilcoxon Signed Rank test p>0.05). In every age group above age 15, clinical vivax disease showed a significant male bias (Figure 2A; Mann–Whitney test p<0.001, Wilcoxon Signed Rank test p<0.03). Falciparum malaria also failed to show a sex bias in children, whether ≤10 or ≤15 years of age (Figure 2C; Mann–Whitney and Wilcoxon Signed Rank test p>0.05). Interestingly, this group did not exhibit an adult male bias when >15 age groups were compared with children ≤15 years of age. However, reducing the age limit of children to ≤10 years resulted in an adult male bias for *P. falciparum* as well. Every age group above age 15 showed a distinct adult male bias (Figure 2C; Mann–Whitney test p<0.01, Wilcoxon Signed Rank test p<0.03 except the >45 age group). This adult male bias observed in our study was independent of social strata, as it was observed in both low-cost public hospitals and in high-cost private medical centers (Table 3). The adult male bias in clinical malaria was also independent of the season as it was equally evident in the wet monsoon as well as the dry non-monsoon months (Figure S1; *P. falciparum* data not shown).

**Table 3:** Sex bias in clinical malaria across data sources.

Medical centers (region)	Children ≤15					Adults >15				
	SPR$ Male	SPR$ Female	χ^2^ statistic	p value	OR#	SPR$ Male	SPR$ Female	χ^2^ statistic	p value	OR#
***P. vivax***										
Low-cost, tertiary (Mumbai)	3.84	3.21	1.18	0.28	1.2	8.64	4.1	148	<0.0001	2.2
High-cost tertiary (Mumbai)	4.35	0	*	0.54	2.8	25.38	10.9	*	<0.0001	2.8
Low-cost primary (Rourkela)	1.33	1.25	0.33	0.56	0.9	2.65	1.44	24.5	<0.0001	1.9
***P. falciparum***										
Low-cost, tertiary (Mumbai)	1.47	0.87	3	0.09	1.7	2.87	1.05	70.2	<0.0001	2.8
High-cost tertiary (Mumbai)	2.17	0	*	1	1.7	3.6	0.37	*	<0.0001	9.9
Low-cost primary (Rourkela)	2.36	3.19	6.2	0.01	0.7	5.27	3.45	27.6	<0.0001	1.6

$ Percent slide-positivity rate.

# Odds ratio.

* Low numbers necessitated the use of the Fisher's exact test.

**Figure 3:**
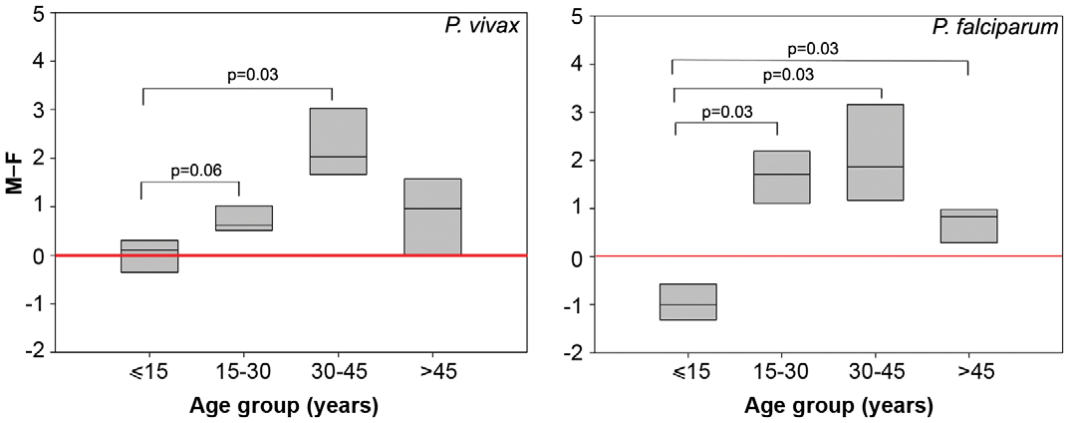
Age distributions of differences between male and female SPRs in the Rourkela clinical malaria dataset. Box plots showing the 25th and 75th percentiles, together with the median, with whiskers showing the minimum and maximum difference in the percent slide-positivity rates between males and females across age groups vivax (A) and falciparum (B) malaria. Data were compared with the difference of male/female SPRs expected under the hypothesis of neutrality (0, red line) and were analyzed with the Mann–Whitney test. Statistically significant values are indicated.

In order to assess whether the parasite species or the mosquito species influenced the observed adult male bias, we examined data from Rourkela, another urban hypoendemic region in Odisha, on the Eastern side of India. While the predominant parasite in Mumbai region is *P. vivax*, in Rourkela it is *P. falciparum*, and instead of *An. stephensi*, malaria is spread by *An. culicifacies* in Rourkela [19]–[22]. We divided the two-year data from the primary health center in Rourkela into four units of six months each to allow for statistical analysis. Analysis of the Rourkela data showed an adult male bias similar to that observed in Mumbai. For vivax malaria, a sex bias was absent in children under 15 (Figure 3). Interestingly, the SPRs of female children were significantly greater than those of male children in the ≤15 age group for falciparum malaria in this region (p<0.01; Figure 3, Table 3). This was in contrast to the data from Mumbai, where the SPRs of male and female children were statistically indistinguishable for both vivax and falciparum malaria. After age 15, however, the SPRs of male patients were significantly greater than those of female patients for both species of the parasite (p<0.0001; Table 3). In the case of falciparum malaria, the SPRs of males were greater than those of females in every age group above age 15. (Figure 3; Mann–Whitney test p<0.03, the two-year data was insufficient for Wilcoxon analysis). Although a trend towards a male bias was observed after age 15 for vivax malaria, it was statistically significant only in the 30–45 age group. Thus, clinical malaria exhibited an adult male bias in both the Mumbai and Rourkela regions.

By contrast, sex bias was absent in the active surveillance dataset, whether for *P. vivax* or for *P. falciparum* (Figure 2B, 2D; p>0.05). The population of individuals tested in active surveillance consisted of about 58% males and 42% females (Table 2). This ratio remained essentially unchanged whether the age group under consideration was children ≤15 years of age (53% male children, 47% female children; Table 2) or adults >15 years of age (59% males, 41% females; Table 2). The p value for Wilcoxon Signed Rank test for every age group in the *P. vivax* active surveillance dataset was >0.05, implying that data was distributed around zero. The Wilcoxon test could not be applied to the *P. falciparum* active surveillance dataset from Mumbai because of the low incidence of the parasite. Essentially similar results were observed when the data was analyzed for seasonal variation (Figure S1; *P. falciparum* data not shown). Field data from the National Institute of Malaria Research suggests the absence of a sex bias in mosquito exposure in Rourkela [unpublished observation, SKS and NV]. Thus our analysis of the active surveillance dataset indicated that the frequency of parasite-positivity in the population was age- and sex-independent.

### Reproductive phases and clinical malaria

**Figure 4:**
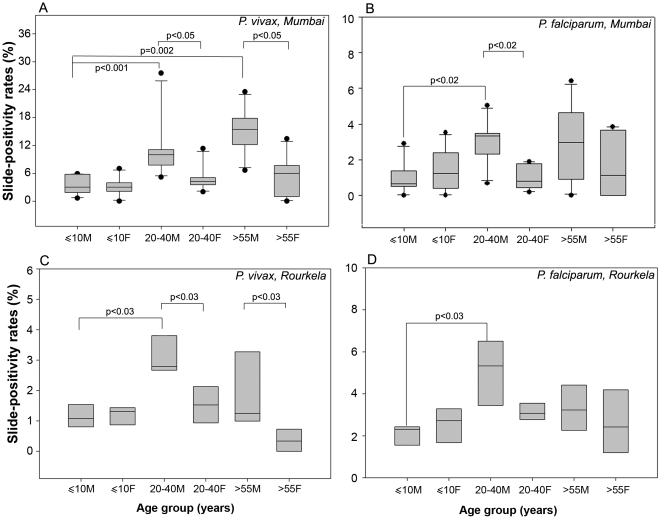
Age distributions of SPRs of males and females in the clinical malaria datasets. A, B, Box plots showing the 25th and 75th percentiles, together with the median, with whiskers showing the minimum and maximum percent slide-positivity rates for males and females across age groups in the Mumbai region for clinical vivax (A) and falciparum (B) malaria. C,D, Box plots as above showing the minimum and maximum percent slide-positivity rates for males and females across age groups in Rourkela for clinical vivax (C) and falciparum (D) malaria. Statistically significant values obtained by the Mann–Whitney test are indicated.

In order to determine if the incidence of clinical malaria was related to puberty or reproductive years (in females), we analyzed the clinical malaria data after regrouping it in ≤10, 20–40, and >55 age bins. The results remained essentially unchanged even when we raised the lower limit of the post-menopausal age bin to 60 years of age and distributed the data in ≤10, 20–40, and >60 age bins (data not shown). We used several methods of analyses to arrive at our conclusions. We compared SPRs of male and females in each age group by Mann–Whitney test. SPR data was also transformed to determine the fraction of positives contributed by males and females in a particular class. The use of such transformed data for statistical analysis ensured that there is no ascertainment bias. We also analyzed the datasets using Chi-square analysis which determined if the proportion of positives were significantly different in the groups under consideration. The Mann–Whitney test of the untransformed data gave the most conservative results, and they are reported in figure 4. Chi-square analysis of the combined data and results of the analysis of transformed data by Mann–Whitney test are given in figures S2 and S3 respectively.

The two parasite species displayed similar patterns of clinical disease, whether in Mumbai or in Rourkela. The incidence of clinical malaria was lowest below the age of 10 in both sexes and was statistically indistinguishable (Figures 4, S2; p>0.05). Post-puberty, the probability of males developing clinical malaria increased dramatically (Figures 4, S2, S3). The female pattern of incidence of clinical disease contrasted starkly with that of males, and it was similar for both parasite species. For women, the incidence of clinical disease remained statistically indistinguishable throughout their life, whether in the pre-pubertal years, post-puberty, or in the post-menopausal years (Figure 4). Thus, women and children experienced a similar incidence of clinical disease, and this incidence was significantly (*p*<0.05) lower than that of adult males (Figures 4, S2, S3).

## Discussion

The relationship between the pattern of age-specific malaria morbidity and malaria transmission intensity has been established [7], [9], [31]–[36]. In areas of high stable transmission, the incidence of clinical malaria peaks between the ages of 1–5 years and then declines rapidly [7], [9], [32], [33]. Where malaria transmission is less intense, the peak age is later in childhood [31], [34]–[36]. High childhood parasite exposure in these areas results in children bearing the brunt of the disease burden and in residents developing immunity to clinical disease prior to sexual maturity. In the hypoendemic regions where this study was situated, we found the annual frequency of parasite detection to be low (range 0.07–4.6%). Such a low incidence implies that adults will be malaria-naïve and will not possess immunity to clinical disease; hence, all ages and sexes should be equally susceptible to clinical malaria. Yet, our observations indicate a significant adult male bias in clinical disease. We used several methods and statistical tests in our analysis. We compared SPRs of males and females across age groups. We transformed the SPR data to determine the fraction of positives contributed by males and females in a particular class. We also analyzed the datasets using Chi-square analysis. The adult male bias was uniformly observed in all methods of analysis. Clinical disease was lower in pre-pubertal children and women, and it was also statistically indistinguishable in these groups. This age-dependent sex bias in clinical disease was observed for both species of the parasite in two different hypoendemic regions infested with two different species of mosquito vectors.

In the hypoendemic Mumbai and Rourkela regions, low levels of exposure to infective mosquitoes predict a first-time exposure to the malarial parasite in adulthood for a large segment of the population. The pattern of clinical disease differed in minor details in the two regions (such as female bias observed in children <15 years of age in falciparum malaria in Rourkela). However, the most noteworthy feature of clinical malaria for both regions was the dramatic increase in clinical disease in males post-puberty. Our analysis indicates that children ≤10, whether male or female, suffered equally from clinical malaria. In males, the onset of puberty coincided with the beginnings of a significantly increased clinical disease. In contrast to the dramatic increase in clinical disease in post-pubertal males, susceptibility to disease in females remained unchanged post-puberty. Menopause also did not appear to have an effect on susceptibility to clinical disease in women by the most conservative interpretation of the data. Thus, we find that women and children suffered equally from clinical disease and were far less likely to suffer from clinical malaria than adult males.

The observed adult male bias was statistically significant, regardless of the method of analysis employed (p<0.05). It also seems to be independent of socio-economic strata. This bias is observed for clinical vivax and falciparum malaria in two distinct hypoendemic regions where the parasite is spread by two different anopheline species. Multiple parasite strains have been shown to co-exist in both Mumbai and Rourkela [22], [37], [38]. Thus, host/parasite genotype is unlikely to be a major contributor to the observed adult male bias.

Puberty is associated with an array of physiological and behavioral changes. Multiple factors, either singly or in combination, could contribute to the observed post-pubertal male bias in this study. We find an increased incidence of clinical malaria with the onset of puberty in males but not females. It is therefore tempting to implicate male sex hormones in this phenomenon. The role of sex hormones in disease susceptibility in human malarial disease remains ambiguous [12], [39], [40]. Studies in the murine model, where malaria-unexposed adult mice are infected with *Plasmodium*, clearly establish testosterone-linked susceptibility and estrogen-linked resistance to malaria [4], [5]. The exact mechanism of the sex hormone-linked dimorphism in murine malarial infections is not completely understood. There is evidence linking the effect of sex hormones to the functioning of the immune system—especially on the balance between pro-inflammatory and regulatory processes—and responses in the liver [4], [5], [41], [42]. Further investigations are needed to determine if indeed testosterone influences susceptibility to clinical malaria in humans.

In most epidemiological assessments in hypoendemic areas, the data for the active and passive surveillance is clubbed and reported as the incidence of malaria [15], [18], [43]. Even though an adult male bias in clinical malaria has been noted in certain hypoendemic parts of the world, the question of sexual dimorphism remains ambiguous because of a failure to investigate differential vector exposure [15]–[18]. Through our active surveillance dataset, we obtained information regarding frequency of parasite-positivity in a largely healthy population, whereas our passive dataset represented the set that was seeking medical intervention. It was not possible to determine what percentage of men, women, and children that were blood-smear positive by active surveillance actually developed clinical malaria because of the governmental policy of immediately treating all detected positive cases with anti-malarial drugs. Active surveillance allows the identification of the fraction of the population that harbors the parasite at the time of data collection and indicates infection rates.

It could be argued that adult males are more likely to be outdoors than adult females, and hence more likely to be bitten by infected mosquitoes. However this seems unlikely. Our analysis of active surveillance data showed that the frequency of parasite-positivity in the population was age- and sex-independent. The biting patterns of malaria vectors in Mumbai and Rourkela are well characterized. Entomological studies have clearly shown that biting activity of Anopheline mosquitoes is sex independent [44]–[47]. The anthropophilic *An. stephensi*, the principal vector in the Mumbai region, bites throughout the night, both indoors and outdoors [19], [48], [49], and the biting pattern is host sex-independent [44]. *An. culicifacies*, the major vector in Rourkela, also bites indoors and outdoors [50]. Therefore an increased outdoor presence of males, and hence increased vector exposure, is less likely to be the reason for enhanced clinical susceptibility. If increased outdoor activity indeed resulted in greater exposure of adult males to mosquito bites, the age and sex distribution patterns of parasite-positivity would be different for the wet season—when people are forced to remain largely indoors—and the dry season when outdoor activities are possible. Our analysis of both clinical malaria and active surveillance datasets for the monsoon–non-monsoon months showed that the distributions were essentially similar during the wet and dry months suggesting an absence of differential vector exposure. However, in the absence of mosquito landing data and vector exposure of the population in question, it is difficult to absolutely resolve this issue.

Other changes in male behavior occurring post-puberty such as alcohol and tobacco consumption [51], could also contribute to the increasing incidence of clinical malaria in post-pubertal males. Beer consumption has been linked to increased attractiveness for *An. gambiens* [52]. Although beer accounts for <5% of the alcohol consumed in India [51] and the mosquito vectors are different, this study raises the possibility that alcohol intake itself could be linked to increased mosquito landing. Equally, chronic alcohol consumption is likely to cause dysfunction of the liver, an organ important in conferring resistance to both liver- and blood-stages of malarial disease [53], [54]. Conversely, lower nutritional status of and prevalence of anemia in post-pubertal females could influence the incidence of clinical malaria in them. Iron deficiency anemia has been shown to be significantly more common in adolescent Indian girls than boys [55]. Anemia has been shown to have a protective effect against clinical malaria in Kenya [56]. Further experimental support is required to assess the role of anemia in the observed lower incidence of clinical malaria in females. Co-infections with worms, gastrointestinal parasites, etc., could potentially also influence the precipitation of clinical disease [57]–[59]. A paucity of data regarding the role of these factors in this process makes it difficult to assess the role of other infections in age- and sex-dependence of malarial susceptibility in hypoendemic regions. A prospective longitudinal study is now being undertaken by us to address some of these issues.

Our conclusions are likely to be confounded by two major factors:

1. Ascertainment bias in the clinical malaria dataset: We think that ascertainment bias is unlikely to influence our analysis for the following reasons. First, the adult male bias is observed across both primary and tertiary medical clinics, in low and high-cost medical centers and in two different regions of the country. Second, the bulk of our data for the Mumbai region was captured from KEM and Kasturba hospitals that have special clinical malaria programs. Physicians at these hospitals examine patients and on the basis of clinical assessment, all suspected clinical malaria patients —irrespective of gender—are tested for parasite presence by trained microscopists. Similarly, all patients approaching the primary malaria clinics in Rourkela are tested for the presence of the parasite. It would have been ideal to ascertain the illnesses of the malaria-negative population in our clinical malaria pool and analyze these for a sex bias, however, we captured our data from centers with special clinical malaria programs and primary malaria clinics, neither of which maintain any records on the nature of the patients' non-malaria illnesses. Sur et al. [17] have analyzed clinical malaria and typhoid in a cohort of patients in the urban slums of hypoendemic Kolkata and found a sex bias in clinical malaria, but not in typhoid. The vector in this region of India is also *An. stephensi*. This suggests that the phenomenon of sex bias in hypoendemic areas may be a general feature for clinical malaria.
2. Socio-economic factors: Differences in the health-seeking behavior of males and females is likely to cofound our analysis. Indeed, our clinical malaria dataset showed a distinct male bias in health-seeking behavior, which is in accordance with reports of male-biased health-seeking behavior in the Indian subcontinent [60], [61]. Alternatively, it is likely that more males suffer from febrile illnesses in India. A recent report on treatment seeking for febrile-illnesses in Odisha, India, shows an absence of sex bias even in the most economically backward strata, suggesting that men and women are equally likely to seek medical help for their fever [62]. In any case, we think that health-seeking behavior is unlikely to affect our conclusion, given that the test statistic we use for our analysis (SPR) represents the fraction of males or females who were positive for malaria in the pool of (male or female) patients approaching medical facilities for their suspected malaria. Even if there was a degree of under-reporting in the clinical malaria dataset due to differences in health-seeking behaviour in the two sexes, without a dimorphism in susceptibility, the malaria-positivity fraction of males and females would not differ significantly. Our results show that among all patients seeking medical help for their malaria-like symptoms, the percentage of females who suffered from clinical malarial disease was significantly less than the percentage of males suffering from clinical malaria.

Furthermore, we used several different methods of statistical analyses to arrive at this conclusion. Apart from comparing SPRs of males and females, we also stratified the SPR data by age to determine the fraction of positives contributed by males and females in the various age groups and then compared these fractions between males and females within the age group by stringent statistical analysis. This allowed us to avoid ascertainment bias because even though the total number of males approaching medical centers was higher than females, the fraction of positives contributed by males in a given positive pool did not depend on the total number of males tested. The Chi square analysis was also used to determine whether the proportion of positives were statistically significantly different in the given groups. In summary, the use of a proportion avoids ascertainment bias as it does not depend on the total number tested in each class (males *vs* females). We also do not think economic factors are a likely source of distortion in our analysis as ratios of male∶female SPRs in the clinical dataset were ∼2∶1, irrespective of whether the dataset under consideration is a low-cost, high-cost, primary, or tertiary medical center.

### Conclusion

Our study indicates the existence of an adult male bias in clinical malaria in hypoendemic regions of India. Women and pre-pubertal children in our study exhibited the lowest risk of clinical malaria; adult males demonstrated the highest risk. This report may have implications in the development of malarial vaccines and the formulation of anti-malarial health strategies. Phase IIa vaccine clinical trials, which are conducted in non-immune adults, may need to account for this adult male bias in clinical disease when interpreting their results. More importantly, this study will help in understanding the epidemiology of malaria-attributed illnesses and in pinpointing the population segment most at risk in hypoendemic regions. Identifying the population that needs to be treated is vital to the effective targeting of public health resources. Spread across diverse regions of the world, almost 1 billion people currently live under unstable- or low-malaria risk [63]. Global warming is projected to result in the spread of malaria to new geographic areas [64], [65], exposing non-immune adult populations to the parasite. Our epidemiological study suggests that adult males are more likely to suffer from malarial symptoms severe enough to seek hospital referral, but both males and females, whether adults or children, are equally likely to harbor the parasite. These results will therefore be relevant in the formulation and implementation of successful global anti-malarial public health strategies and educational and eradication campaigns.

## Supporting Information

Figure S1 **Age distributions of differences between male and female SPRs in the clinical malaria and active surveillance datasets in the Mumbai region during the Monsoon and Non-monsoon seasons.** A,C, Box plot showing the 25th and 75th percentiles, together with the median, with whiskers showing the minimum and maximum difference in the percent slide-positivity rates between males and females across age groups in the vivax clinical malaria dataset for Monsoon (A) and Non-monsoon (C) season. B,D, Box plots as in A showing the difference in the percent slide-positivity rates between males and females across age groups who tested positive for *P. vivax* in the Monsoon (B) and Non-monsoon (D) season in the active surveillance programme. Data were compared with the difference of male/female SPRs expected under the hypothesis of neutrality (0, red line) and were analyzed with the Mann–Whitney test. Statistically significant values are shown in black. Numbers in red indicate statistically significant p values obtained by Wilcoxon Signed Rank test under the hypothesis that the median of the group did not differ significantly from zero. The test could not be applied to the falciparum data in the active surveillance dataset.
(TIF)

Figure S2 **Age distributions of SPRs of males and females in the combined clinical malaria datasets.** A, B, Percent slide-positivity rate of *P. vivax* (A) and *P. falciparum* (B) attributable clinical disease amongst male (grey bars, M) and female (black bars, F) patients across age groups in the combined Mumbai dataset. C, D, Percent slide-positivity rate of *P. vivax* (C) and *P. falciparum* (D) attributable clinical disease amongst male (grey bars, M) and female (black bars, F) patients across age groups in the combined Rourkela dataset. Statistically significant values obtained by Chi-square analysis between the sexes for a given age group and across age group within the same sex are indicated.
(TIF)

Figure S3 **Fraction contributed by males and females to the total positive pool in a particular age group in clinical malaria datasets.** A, B, Box plots showing the 25th and 75th percentiles, together with the median, with whiskers showing the minimum and maximum fraction contributed to the total positive pool by males (M) and females (F) across age groups in the Mumbai region for clinical vivax (A) and falciparum (B) malaria. C, D, Box plots showing the 25th and 75th percentiles, together with the median, with whiskers showing the minimum and maximum fraction contributed to the total positive pool by males (M) and females (F) across age groups in the Rourkela region for clinical vivax (C) and falciparum (D) malaria. Statistically significant values obtained by the Mann–Whitney test are indicated.
(TIF)
