# Supplementary figures and images for: Age-Dependent Sex Bias in Clinical Malarial Disease in Hypoendemic Regions
Source: PLoS One. 2012 Apr 25;7(4):e35592. doi: 10.1371/journal.pone.0035592 (PMC3338423; doi:10.1371/journal.pone.0035592)

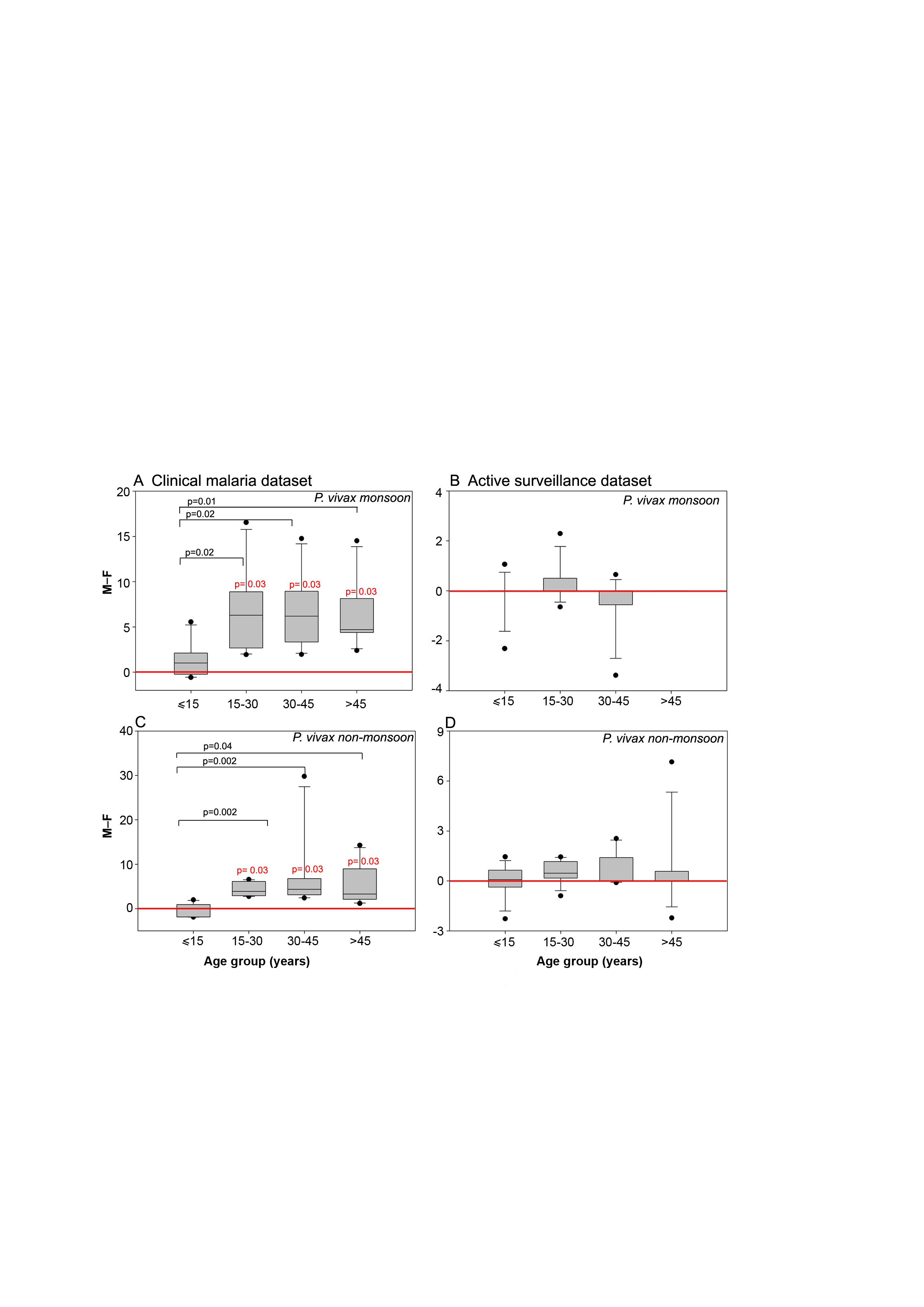

Supplement: Figure S1 — Age distributions of differences between male and female SPRs in the clinical malaria and active surveillance datasets in the Mumbai region during the Monsoon and Non-monsoon seasons. A,C, Box plot showing the 25th and 75th percentiles, together with the median, with whiskers showing the minimum and maximum difference in the percent slide-positivity rates between males and females across age groups in the vivax clinical malaria dataset for Monsoon (A) and Non-monsoon (C) season. B,D, Box plots as in A showing the difference in the percent slide-positivity rates between males and females across age groups who tested positive for P. vivax in the Monsoon (B) and Non-monsoon (D) season in the active surveillance programme. Data were compared with the difference of male/female SPRs expected under the hypothesis of neutrality (0, red line) and were analyzed with the Mann–Whitney test. Statistically significant values are shown in black. Numbers in red indicate statistically significant p values obtained by Wilcoxon Signed Rank test under the hypothesis that the median of the group did not differ significantly from zero. The test could not be applied to the falciparum data in the active surveillance dataset. (TIF) [file pone.0035592.s001.tif]

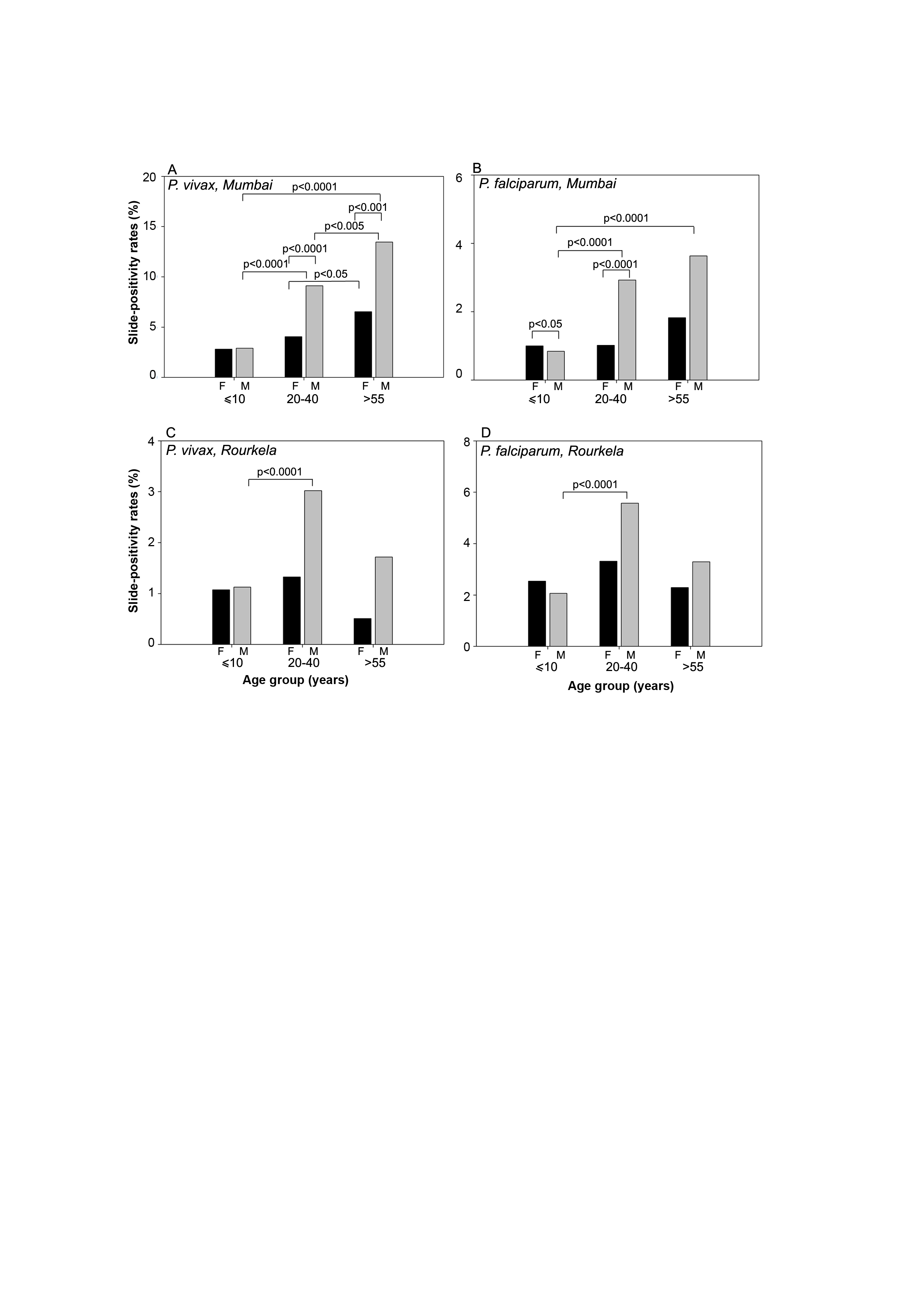

Supplement: Figure S2 — Age distributions of SPRs of males and females in the combined clinical malaria datasets. A, B, Percent slide-positivity rate of P. vivax (A) and P. falciparum (B) attributable clinical disease amongst male (grey bars, M) and female (black bars, F) patients across age groups in the combined Mumbai dataset. C, D, Percent slide-positivity rate of P. vivax (C) and P. falciparum (D) attributable clinical disease amongst male (grey bars, M) and female (black bars, F) patients across age groups in the combined Rourkela dataset. Statistically significant values obtained by Chi-square analysis between the sexes for a given age group and across age group within the same sex are indicated. (TIF) [file pone.0035592.s002.tif]

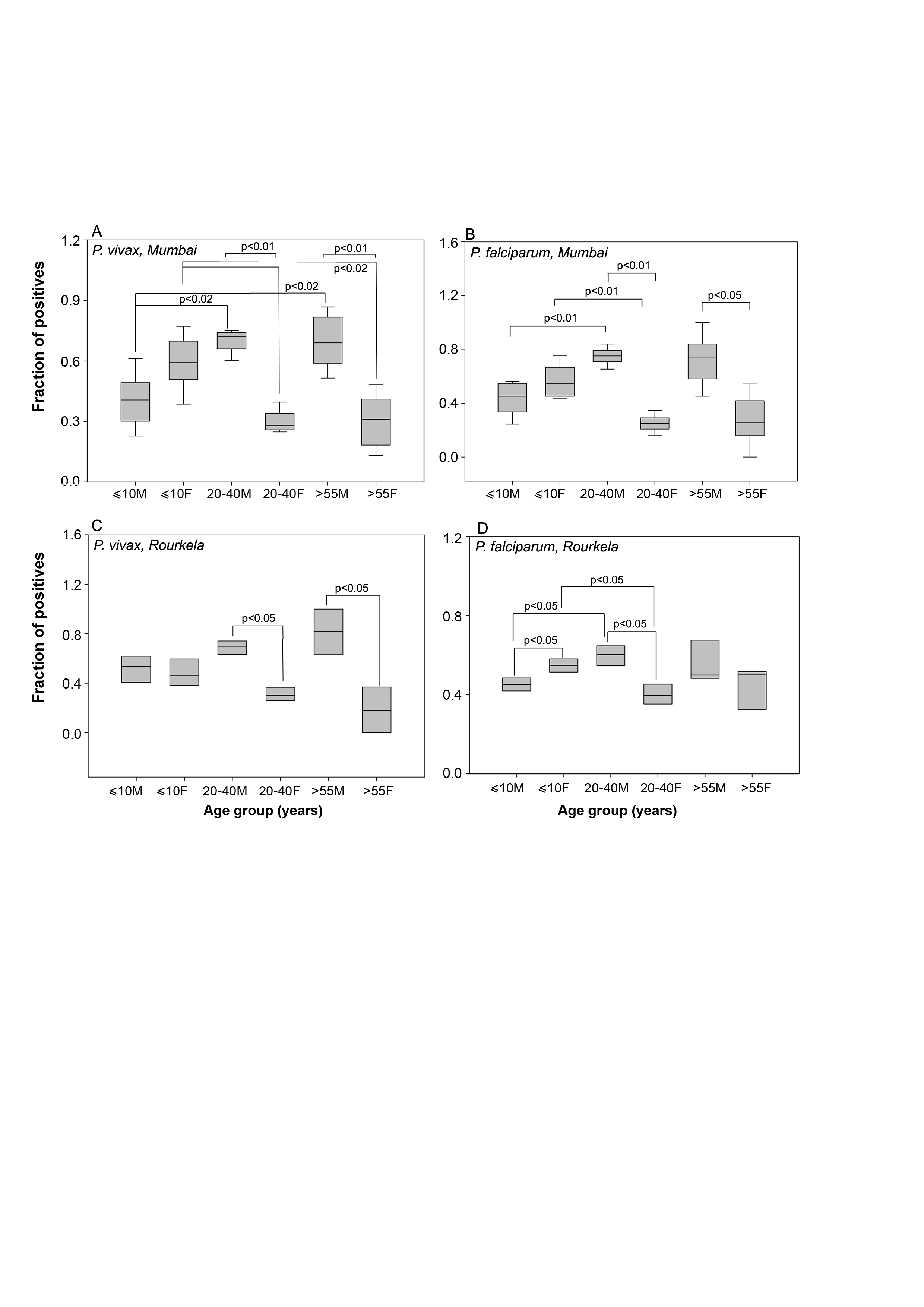

Supplement: Figure S3 — Fraction contributed by males and females to the total positive pool in a particular age group in clinical malaria datasets. A, B, Box plots showing the 25th and 75th percentiles, together with the median, with whiskers showing the minimum and maximum fraction contributed to the total positive pool by males (M) and females (F) across age groups in the Mumbai region for clinical vivax (A) and falciparum (B) malaria. C, D, Box plots showing the 25th and 75th percentiles, together with the median, with whiskers showing the minimum and maximum fraction contributed to the total positive pool by males (M) and females (F) across age groups in the Rourkela region for clinical vivax (C) and falciparum (D) malaria. Statistically significant values obtained by the Mann–Whitney test are indicated. (TIF) [file pone.0035592.s003.tif]
